# An Approach to Identify New Pleiotropic Genetic Loci From Publicly Available Univariate GWAS Results

**DOI:** 10.1093/geroni/igaa057.426

**Published:** 2020-12-16

**Authors:** Yury Loika, Alexander Kulminski

**Affiliations:** Duke University, Durham, North Carolina, United States

## Abstract

The connections between genes and multifactorial polygenic age-related traits are not trivial due to complexity of metabolic networks in an organism, which were primarily adapted to maximize fitness at reproductive age in ancient environments. Given this complexity, pleiotropy in predisposition to complex traits appears to be common phenomenon. Identifying mechanisms of pleiotropic predisposition to multiple age-related traits can be a key factor in developing strategies for extending health-span and lifespan. Correlation between complex traits may be a factor shedding light on these mechanisms. Recently, we used an omnibus test leveraging correlation between multiple age-related traits to gain insights into pleiotropic predisposition to them. The analysis using individual-level data identified large number of new pleiotropic loci and highlighted a novel phenomenon of antagonistic genetic heterogeneity, which was characterized by antagonistic directions of genetic effects for directly correlated traits. Here, we demonstrate feasibility of our approach using summary statistics from univariate genome-wide (GW) association studies (GWAS). Our analysis focused on the results for high density lipoprotein cholesterol (HDL-C) and triglycerides (TG) from the Global Lipids Genetic Consortium, which reported 94 GW significant loci (p≤5×10-8). The traits’ correlation was estimated from the individual level data. Our approach identified 28 loci with pleiotropic predisposition to HDL-C and TG at p≤5×10-8, which did not attain univariate GW significance with either of these traits. Fifteen of them (53%) demonstrated antagonistic heterogeneity. These results show that our approach can be efficiently used in the analysis of summary statistics from published studies to identify novel pleiotropic loci.

